# Circulating Irisin in Children and Adolescents With Prader-Willi Syndrome: Relation With Glucose Metabolism

**DOI:** 10.3389/fendo.2022.918467

**Published:** 2022-06-14

**Authors:** Stefania Mai, Danilo Fintini, Chiara Mele, Alessio Convertino, Sarah Bocchini, Graziano Grugni, Gianluca Aimaretti, Roberta Vietti, Massimo Scacchi, Antonino Crinò, Paolo Marzullo

**Affiliations:** ^1^ Laboratory of Metabolic Research, Istituto Auxologico Italiano, Istituto di Ricovero e Cura a Carattere Scientifico (IRCCS), San Giuseppe Hospital, Piancavallo, Verbania, Italy; ^2^ Reference Center for Prader Willi Syndrome, Research Institute, Bambino Gesù Children’s Hospital, Rome, Italy; ^3^ Division of Endocrinology, Department of Translational Medicine, University of Piemonte Orientale, Novara, Italy; ^4^ Division of Auxology, Istituto Auxologico Italiano, IRCCS, San Giuseppe Hospital, Piancavallo, Verbania, Italy; ^5^ Department of Clinical Sciences and Community Health, University of Milan, Milan, Italy

**Keywords:** irisin, PWS, obesity, glucose metabolism, children, adolescents

## Abstract

Irisin is a myokine involved in the browning of white adipose tissue and regulation of energy expenditure, glucose homeostasis and insulin sensitivity. Debated evidence exists on the metabolic role played by irisin in children with overweight or obesity, while few information exist in children with Prader Willi Syndrome (PWS), a condition genetically prone to obesity. Here we assessed serum irisin in relation to the metabolic profile and body composition in children and adolescents with and without PWS. In 25 PWS subjects [age 6.6-17.8y; body mass index standard deviation score (BMI SDS) 2.5 ± 0.3] and 25 age, and BMI-matched controls (age 6.8-18.0y; BMI SDS, 2.8 ± 0.1) we assessed irisin levels and metabolic profile inclusive of oral glucose tolerance test (OGTT), and body composition by dual-energy X-ray absorptiometry (DXA). In PWS, we recorded lower levels of fat-free mass (FFM) (p <0.05), fasting (p<0.0001) and 2h post-OGTT insulin (p<0.05) and lower insulin resistance as expressed by homeostatic model of insulin resistance (HOMA-IR) (p<0.0001). Irisin levels were significantly lower in PWS group than in controls with common obesity (p<0.05). In univariate correlation analysis, positive associations linked irisin to insulin OGTT_0_ (p<0.05), insulin OGTT_120_ (p<0.005), HOMA-IR (p<0.05) and fasting C-peptide (p<0.05). In stepwise multivariable regression analysis, irisin levels were independently predicted by insulin OGTT_120_. These results suggest a link between irisin levels and insulin sensitivity in two divergent models of obesity.

## Introduction

Prader-Willi Syndrome (PWS) is a rare genetic disease caused by the lack of expression of paternal genes on chromosome 15q11.2-q13 (1) and it represents one of the most common forms of genetic obesity. The estimated incidence rate is 1 in 25,000 live births (2). Three main genetic mechanism have been detected in PWS: interstitial deletion of the proximal long arm of chromosome 15 (15q11-q13)(DEL15), maternal uniparental disomy of chromosome 15 (UPD15) and imprinting defects (3).

PWS is a complex multisystem disorder, characterized by infantile lethargy and muscle hypotonia followed by hyperphagia and excess weight gain during early child-hood, mildly dysmorphic acro-facial features, kyphoscoliosis, developmental delay with learning and behavioral problems, and a variable number of hypothalamo-pituitary disorders comprising impaired GH secretion with short stature, hypogonadism, hypothyroidism (1, 2, 4, 5). A typical feature of PWS is a lack of satiety due to hypothalamic dysfunction, which generates obsessive craving for food approximately by the age of 2 years and progresses to development of severe obesity in a large proportion of patients unless overeating is promptly controlled by caregivers (6).

With respect to subjects with common obesity, obese children with PWS display distinct phenotypic and metabolic features (1), consisting of higher fat mass (FM), lower fat-free mass (FFM) and impaired muscle function (7, 8). Despite this adverse phenotype, fasting insulin levels and insulin resistance are comparatively lower at all ages in obese patients with PWS (9–11). Furthermore, non-obese children with PWS showed lower insulin and glucose levels than their obese counterpart (12). When plotted against results obtained in non-PWS controls, reasons for higher insulin sensitivity in PWS include a modest accumulation of visceral fat after adjustment for total adiposity (13), an disproportionately elevated ghrelin concentration for the degree of obesity (8, 14), high levels of adiponectin (10) and an impaired GH secretion (15).

In the past few years, attention has focused on irisin, a myokine involved in the cross-talk between muscle and adipose tissue (16, 17) that has been proposed to play a role in the pathophysiology of obesity. Originally described in mice by Böstrom et al. (16), irisin is a 112 amino acid cleavage product of fibronectin type III domain-containing protein 5 (FDNC5), that was shown to stimulate the “browning” of white adipocytes, thus acting to increasing total body energy expenditure, reducing body weight, and reducing insulin resistance (16, 18). While irisin was first identified as a myokine secreted in response to exercise, recent studies in rodents and humans demonstrated that irisin is also expressed and secreted by white adipose tissue (WAT) (17), and there is emerging evidence that irisin could also act as an adipokine (17, 19, 20). As such, in the adult population circulating irisin is positively associated with body weight (21–24) and several measures of adiposity (22, 23), as well as muscle mass (20–22).

On the contrary, scanty and debated information exists on the role played by irisin in relation to pediatric obesity (25). While studies found a positive correlation between irisin and body mass index (BMI), waist circumference (WC) and fat-free mass (26–28), others documented a negative or a lack of correlation between irisin and adiposity measures (29–31). Conceivably, at this stage divergences may depend on variability in hormonal activity, muscle development and body fat accumulation in growing children (32, 33).

With regard to irisin and glucose metabolism, its role is not completely understood both in children and adults (18, 25). Irisin facilitates glucose uptake by skeletal muscle and adipose tissue (by increasing the expression of GLUT4) and improves hepatic glucose metabolism, increasing glycogenesis and reducing gluconeogenesis (18). Also, it contributes to β-islet cell survival and function (34). In obese children, irisin concentrations have been correlated to glucose and insulin levels as well as insulin resistance (27, 28,31, 35). Intriguingly, a cross-sectional study in obese children with and without insulin resistance recorded no difference in serum irisin levels (36), while a negative correlation was observed in healthy children between fasting glucose and irisin levels (37).

In PWS, circulating irisin level have only been assessed in a few studies (20, 38, 39). A previous study from our group in adult obese subjects with PWS reported significantly lower irisin levels in PWS than in controls with common obesity, while being similar to values recorded in lean subjects (20). In a recent study encompassing children and adults, Faienza et al. showed that serum irisin levels didnot differ between PWS and normal weight subjects but, interestingly, the authors found significantly lower irisin levels both in pediatric and adult PWS subjects carrying DEL15 as compared to controls (40).

Given this background, our study aimed to explore circulating irisin in relation to the metabolic profile and body composition in obese children and adolescents with and without PWS. Secondary aim of the study was to assess the predictors of irisin levels in our cohorts.

## Materials and Methods

### Patients

This study enrolled 50 patients, consisting of 25 PWS children and adolescents (16 M/9 F; age, 6.6-17.8y; BMI SDS, 2.5 ± 0.3) and 25 age, gender and BMI-matched control subjects (11 M/14 F; age, 6.8-18.0y; BMI SDS, 2.8 ± 0.1). PWS subjects were referred to the Center for Prader-Willi Syndrome of the Bambino Gesù Children’s Hospital in Rome, Italy. All PWS individuals received a diagnosis based on typical syndromic features confirmed by molecular genetic studies of chromosome 15, including 15q11-q13 deletion in 15 (8 males and 7 females) and UPD15 in the remaining 10 patients (8 males and 2 females).

With respect to hormone replacement therapy, 16 patients with PWS were treated with growth hormone, while 1 PWS patient and one control subject were treated with levothyroxine. For all study participants, exclusion criteria included previously known liver disease, kidney failure, autoimmune diseases, uncontrolled hypothyroidism and/or diabetes mellitus, chronic exposure to anti-inflammatory steroids. Alcohol consumption was investigated, and none was an alcohol drinker. The study design was conformed to the ethical guidelines of the Declaration of Helsinki (1975) and was firstly approved by the Ethical Research Committees of the Bambino Gesù Children’s Hospital (protocol 2092/2020 approved 25 march 2020).

Written informed consent was obtained from all participants by their parents, and from patients, when appropriate. The study protocol was conformed to the guidelines of the European Convention on Human Rights and Biomedicine concerning biomedical research.

### Body Measurements

Weight and height were measured to the nearest 0.1 kg and 0.1 cm, respectively, using standard methods. Waist circumference (WC) was measured midway between the lowest rib and the top of the iliac crest after expiration; hip measurements were taken at the greatest circumference around the nates. BMI was calculated as weight in kg divided by the square of the height in meters and expressed as standard deviation score, and BMI SDS was calculated according to BMI reference tables (41). The BMI cut-of point of ≥ 2 SDS was used to define obesity (41). Pubertal development was assessed according to Tanner’s criteria (42). A dual-energy X-ray absorptiometry using a Hologic QDR Discovery, and the APEX-system software version 13.3 (Hologic Bedford, MA) with fan beam in array mode was performed by the same operator. Quality control scans were performed daily using a simulated L1-4 lumbar spine phantom. The measurements were performed by using standard positioning techniques. Total body scans were obtained to estimate Fat Mass (FM%), Fat Free Mass (FFM%) expressed as percentage of total body weight (total body less head, with the skull excluded from analysisis). Trunk Fat (TFM), and FFM/FM ratios were also calculated. Coefficient of variation were between 0.3285 to 1.038%.

### Metabolic Studies

Glucose homeostasis was evaluated by fasting glucose levels, oral glucose tolerance test (OGTT)-derived glucose and insulin levels at time 0 and 120 min, and glycated hemoglobin (HbA1c) levels in all subjects. Glucose tolerance was assessed according to ADA guidelines for children and adolescents (43). Insulin resistance was calculated by the homeostatic model of insulin resistance (HOMA-IR) index: insulin (mIU/L) ×[glucose (mmol/L)/22.5] (44). Blood samples were drawn at 08.00 a.m. under 12 hours fasting conditions then vials were centrifuged, and sera were stored at − 80°C until requested. Serum irisin levels were assessed using a commercially available human ELISA kit EK-067–29 (Phoenix Pharmaceutics, Inc, Burlingame, CA, USA) in accordance with the manufacturer’s instructions. This ELISA is specific for human irisin, and quality controls were included in all ELISA measurements with the results falling within the expected range. All samples were analyzed in duplicate. Intra-assay and inter-assay coefficients of variation (CV) of irisin immunoassays were less than 10% and 15% respectively, and minimum detectable concentration was 1.5 ng/mL. Serum leptin concentrations were quantified using a commercially available ELISA kit (Mediagnost GmbH, Reutlingen, Germany) with overall inter- and intra-assay CVs of 6.8–8.3% and 5.5–6.9% respectively.

Serum adiponectin levels were determined by an enzyme linked immunosorbent assay (DRG Instruments GmbH, Marburg, Germany), the detection limit was 1.56–100 ng/ml, sensitivity was 0.2 ng/ml, inter- and intra-assay CV was 2.4–8.4 and 0.9–7.4%, respectively.

Routine laboratory data included levels of aspartate aminotransferase (AST), alanine aminotransferase (ALT), gamma-glutamyl transpeptidase (GGT), glucose, total cholesterol (CHO), high-density (HDL) and low-density lipoprotein (LDL) cholesterol, triglycerides (TG) and HbA1c, measured by enzymatic methods (Roche Diagnostics, Mannheim, Germany). Levels of insulin were measured using a Cobas Integra 800 Autoanalyzer (Roche Diagnostics, Indianapolis, IN, USA). A two-site, solid-phase chemiluminescent immunometric assay or competitive immunoassay (Immulite 2000 Analyzer; DPC, Los Angeles, CA) was used to determine C-peptide levels.

### Statistical Analysis

Statistical analysis was performed using SPSS version 21 (Somers, NY, USA). Values are expressed as means ± standard error mean (SEM). Data points not normally distributed obtained by the Shapiro–Wilk test were log-transformed to improve the symmetry and homoscedasticity of the distribution. For comparative analysis, ANOVA between the 2 groups was used. Pearson’s correlation analysis was used to identify significant associations between variables of interest. Stepwise multivariable regression analysis was used to evaluate the independent association of irisin with metabolic, anthropometric or biochemical parameters.

Two multilinear models were built which included the obese phenotype (common obesity = 0; PWS = 1) in association with parameters of body composition and metabolism (model 1: group, BMI SDs; Glucose OGTT_0_, Glucose OGTT_120_, Insulin OGGT_0_, Insulin OGTT_120_ C-Peptide, HOMA-IR; leptin, adiponectin; model 2: group, BMI SDS, Glucose OGTT_0_, Glucose OGTT_120_, Insulin OGTT_0_, C-Peptide, HOMA-IR, leptin adiponectin; with the exclusion of predictor from the previous model: Insulin OGTT_120_). β coefficients and significance values obtained from the regression models are reported. A p value < 0.05 was considered as statistically significant.

## Results

A summary of anthropometric and biochemical data is reported in **Tables 1**, **2**. BMI standard deviation score (SDS) values were comparable between the two groups and ranged, collectively, between 1.1 and 5.7. Within the PWS group, 14 out of 25 were obese (BMI > 2 SDS) and, overall, 15 were prepubertal and 10 were postpubertal. Among controls, 22 out of 25 were obese and, overall, 14 were prepubertal and 11 were postpubertal. No difference in age and gender distribution were observed between groups.

**Table 1:** Summary of anthropometric data obtained in PWS patients and controls.

Variables	PWS patients	Controls	P value
	(n=25)	(n=25)	
Males/females	16/9	11/14	0.16
Prepubertal/pubertal	15/10	14/11	0.78
Age (years)	11.1 ± 0.6	12.60 ± 0.7	0.16
BMI SDS	2.5 ± 0.3	2.8 ± 0.1	0.31
Weight (kg)	**51.9 ± 3.2**	**71.8 ± 3.5**	**<0.0001**
Height (cm)	**139.0 ± 2.5**	**155.7 ± 3.0**	**<0.0001**
Waist (cm)	**82.3 ± 2.9**	**95.9 ± 2.5**	**0.002**
Hip (cm)	**92.1 ± 2.7**	**102.1 ± 2.3**	**0.013**
Waist-to-hip ratio	**0.89 ± 0.01**	**0.94 ± 0.01**	**0.04**
FM (%)	47.2 ± 4.6	43.6 ± 4.61	0.12
FM (Kg)	28.5 ± 2.1	32.1 ± 2.0	0.31
FFM (%)	50.7± 4.9	53.3± 5.0	0.20
FFM (kg)	**29.8± 1.8**	**39.9± 2.6**	**0.014**
Trunk fat (kg)	12.1± 1.1	14.2± 1.0	0.262
FFM/FM ratio	1.11± 0.05	1.29± 0.1	0.219

Data are expressed as mean ± SEM. Comparison between populations was performed by ANOVA test. Significant differences are shown in bold characters.

BMI SDS, body mass index standard deviation score FM, fat mass; FFM, fat-free mass.

**Table 2:** Summary of biochemical data obtained in PWS subjects and controls.

Variables	PWS (n=25)	Controls (n=25)	P value
Glucose OGTT_0_ (mg/dL)	83.3. ± 1.5	83.8. ± 1.8	0.84
Glucose OGTT_120_ (mg/dL)	105.2 **± 5**.6	107.5 ± 5.6	0.78
Insulin OGTT_0_ (mIU/L)	**10.1 ± 1.0**	**19.0 ± 1.5**	**<0.0001**
Insulin OGTT_120_ (mIU/L)	**39.8 ± 4.4**	**112.8 ± 15.1**	**0.006**
C-Peptide (μg/L)	**1.4 ± 0.1**	**2.1 ± 0.1**	**<0.0001**
HbA1c (%)	5.1 ± 0.3	5.0 ± 0.1	0.35
HOMA-IR	**2.1 ± 0.3**	**4.0 ± 0.4**	**<0.0001**
AST (U/L)	24.6 ± 1.6	24.6 ± 2.1	0.99
ALT (U/L)	23.3 ± 3.2	26.7 ± 4.6	0.55
GGT (U/L)	15.3 ± 1.2	15.4 ± 1.4	0.94
TG (mg/dL)	80.4 ± 9.8	95.4 ± 8.2	0.25
Tot CHO (mg/dL)	165.6 ± 6.1	164.5 ± 6.7	0.91
LDL CHO (mg/dL)	96.1. ± 4.0	96.3 ± 6.0	0.98
HDL CHO (mg/dL)	52.6 ± 2.1	47.4 ± 2.2	0.095
Irisin (ng/mL)	**22.4 ± 0.7**	**25.7 ± 1.1**	**0.013**
Leptin (ng/ml)	31.2 ± 3.7	27.9 ± 2.0	0.42
Adiponectin (µg/ml)	**15.2 ± 1.5**	**9.5 ± 0.9**	**0.001**

Data are expressed as mean ± SEM. Comparison between populations was performed by ANOVA test. Significance is shown in bold characters.

OGTT, Oral Glucose Tolerance Test; OGTT_0_ and OGTT_120_, OGTT at 0 and 120 min; HbA1c, glycated haemoglobin; HOMA-IR, homeostatic model of insulin resistance; AST, aspartate aminotransferase; ALT, alanine aminotransferase; GGT, gamma glutamyl transpeptidase; TG, triglycerides CHO, total cholesterol; LDL CHO, low density lipoprotein cholesterol; HDL CHO, high density lipoprotein cholesterol.

Following OGTT, abnormal glucose metabolism was detected in 2 children with PWS (8%) and 4 controls (16%): impaired fasting glucose was found in 1 subject of the control group and no patient with PWS while impaired glucose tolerance was found in 2 patients with PWS and 3 controls. No cases of type 2 diabetes mellitus (T2DM) were diagnosed in either group.

Anthropometric parameters differed between groups, and subjects with PWS showed lower values of FFM (p <0.05), WC (p <0.005) and waist-to-hip ratio (p<0.05) with respect to controls.

Children and adolescents with PWS harbored lower insulin OGTT_0_ (p <0.0001) and insulin OGTT_120_ levels (p<0.05) than controls. PWS group also showed lower fasting C-peptide levels (p<0.0001), lower insulin resistance expressed as HOMA-IR (p<0.0001) and higher adiponectin levels (p<0.005) compared to controls (**Table 2**). There were no differences in lipids and liver enzymes between populations.

**Figure 1:**
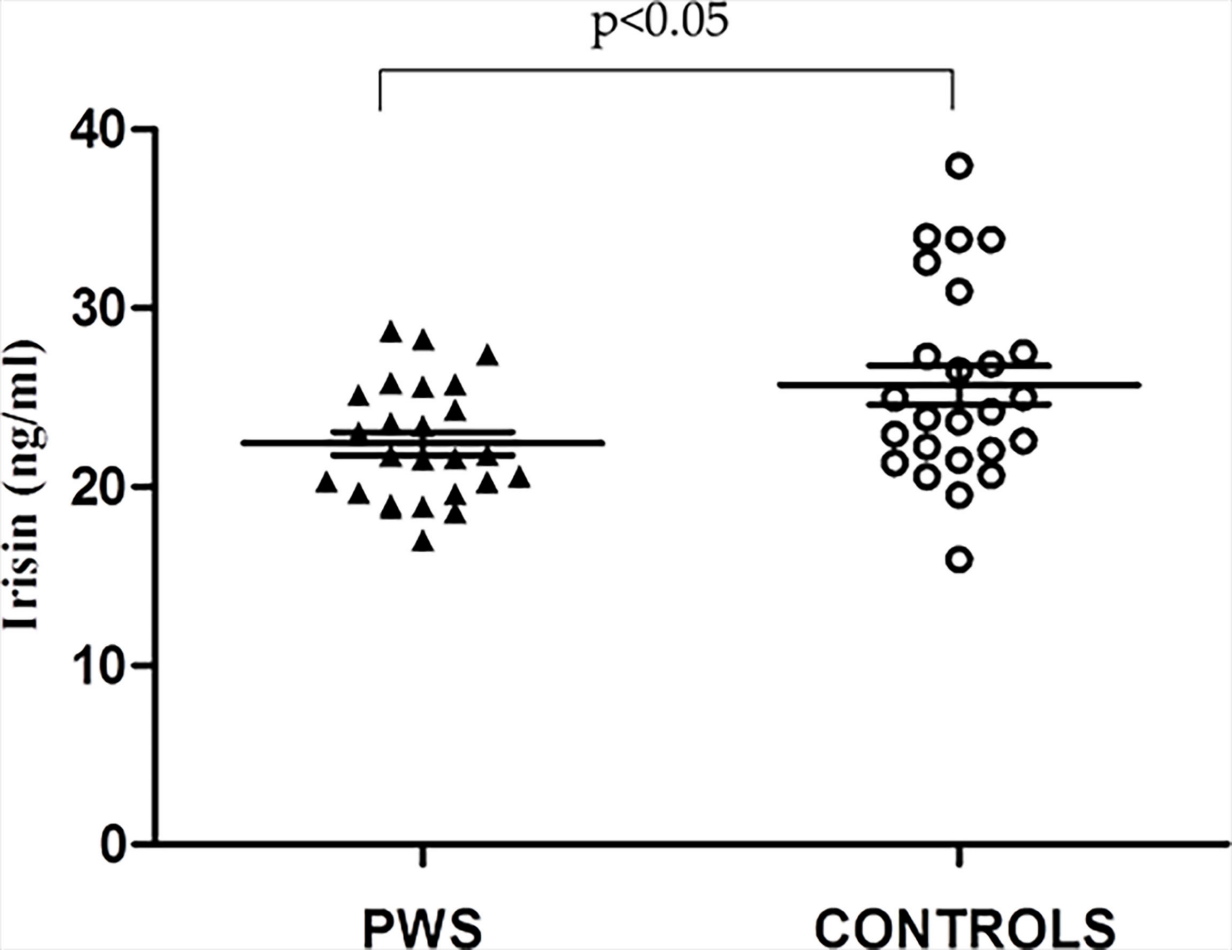
Individual values of circulating irisin levels obtained in Prader Willi Syndrome (PWS) patient (black triangles) and controls (white circles). Lines represent mean ± standard error of the mean (SEM) values.

Analysis of circulating irisin showed measurable levels in all cases. As illustrated in **Figure 1**, the distribution of irisin levels was somewhat more dispersed around the mean in patients with PWS than controls, with the former exhibiting lower concentrations of irisin. When only obese subjects were compared, mean circulating irisin was also significantly lower in the subgroups of obese PWS patients that in obese controls (21.8 ± 1.6 vs 25.9 ± 1.1 ng/ml, p<0.05). In more detailed analysis, irisin levels of PWS patients with DEL15 were significantly reduced compared with controls with common obesity (21.8 ± 0.8 ng/ml and 25.8 ± 1.1 ng/ml, p=0.018) while PWS patients with UPD15 did not show significant differences compared with controls (23.3 ± 1.0 ng/ml and 25.7 8 ± 1.1 ng/ml, p=0.2).

In gender-based analysis, irisin levels did not differ between males and females in PWS (22.08 ± 0.8 and 23.07 ± 1.1 ng/ml, p =0.5) and controls (27.1 ± 2.4 and 24.0 ± 1.4 ng/ml p=0.07). Analysis by pubertal stage showed no difference in irisin levels between prepubertal and postpubertalsubjects both in the PWS (22.1 ± 0.8 vs 22.9 ± 1.1, p=0.5) and control group (26.4 ± 1.5 vs 24.9 ± 1.1, p=0.5).

Correlation analysis in separate groups showed no significant association between irisin and the study variables. In merged datasets (**Table 3**), positive associations linked irisin to PWS status, insulin OGTT_0_ and insulin OGTT_120_ levels, HOMA-IR and fasting C-peptide levels.

**Table 3:** Pearson’s correlation analysis between irisin levels and anthropometric and biochemical parameters in the study populations as a whole.

Parameters	Irisin levels	
	r	P value
Age	0.166	0.25
PWS status	**-0.35**	**0.013**
BMI SDS	- 0.06	0.69
FM (%)	- 0.10	0.56
FM (kg)	0.01	0.63
FFM (kg)	0.22	0.20
FFM (%)	0.10	0.60
Glucose OGTT_0_ (mg/dL)	0.18	0.20
Glucose OGTT_120_ (mg/dL)	0.11	0.47
Insulin OGTT_0_ (mIU/L)	**0.30**	**0.042**
Insulin OGTT_120_ (mIU/L)	**0.58**	**0.004**
HOMA-IR	**0.30**	**0.045**
C- Peptide (μg/L)	**0.30**	**0.034**
HbA1_C_ (%)	-0,084	0,57
Leptin (ng/ml)	0.17	0.25
Adiponectin (µg/ml)	- 0.201	0.16

For PWS status: PWS = 1, obese control = 0. Significance is shown in bold characters.

BMI SDS, body mass index standard deviation score; FM, fat mass; FFM, fat-free mass. OGTT, Oral Glucose Tolerance Test; OGTT_0_ and OGTT_120_, OGTT at 0 and 120 min; HbA1c, glycated haemoglobin; HOMA-IR, homeostatic model of insulin resistance.

After controlling for age, sex and BMI SDS, correlation remained significant between irisin and insulin OGTT_0_ (r=0.345, p<0.05), insulin OGTT_120_ (r=0.622, p<0.005), HOMA-IR (r= 0.338, p<0.05) and C-peptide (r=0.325, p<0.05). When controlled for groups (PWS and non-PWS), only the correlation between irisin and insulin OGTT_120_ was maintained (r = 0.45, p= 0.041).

Stepwise multivariable regression analysis was performed in the two groups as a whole, and a number of models were explored to analyze the predictors of irisin levels in the study populations. As shown in **Table 4**, analysis documented that irisin levels were only predicted by insulin OGTT_120_ levels (β =0.58, p=0.005). After its removal from the regression equation, the PWS group acted as independent negative predictor of irisin levels (β =-0.35, p=0.025). No variable entered the regression equation in analyses on separate groups.

**Table 4:** Regression coefficients derived from the stepwise multivariable regression analysis conducted in the PWS subjects and obese controls as a whole on irisin as the dependent variable.

Multivariable regression analysis	Included variables			Excluded variables
		β	P value	
**Model 1**	Insulin OGTT_120_	0,58	0,005	group, BMI SDS, Glucose OGTT_0_, Glucose OGTT_120_, Insulin OGTT_0_, C-Peptide, HOMA-IR, leptin, adiponectin
**Model 2** (Insulin OGTT _120_ not included)	Group	-0,35	0,025	BMI SDS, Glucose OGTT_0_, Glucose OGTT_120,_ Insulin OGTT_0_, C-Peptide, HOMA-IR, leptin, adiponectin.

Group (PWS = 1, obese controls = 0) was introduced as independent variable in all models. Other independent variables introduced in the two models: model 1: group, BMI SDS, Glucose OGTT0, Glucose OGTT120, Insulin OGTT_0_, Insulin OGTT_120_, C-Peptide, HOMA-IR, leptin, adiponectin; model 2: BMI SDS, Glucose OGTT0, Glucose OGTT_120_, Insulin OGTT_0_, C-Peptide, HOMA-IR, leptin, adiponectin. β standardized coefficients and p values are shown.

HOMA-IR, homeostatic model of insulin resistance; OGTT, Oral Glucose Tolerance Test; OGTT_0_ and OGTT_120_, OGTT at 0 and 120 min.

## Discussion

The present study analyzed circulating irisin levels in children and adolescents with and without PWS in relation to metabolic profile and body composition. Current results show that irisin levels are lower in PWS than in controls independent of BMI, and that a strong association links irisin to measures of insulin resistance, particularly insulin OGTT_120_ levels.

It is well known that clinical features of PWS include muscle hypotonia, endocrine disturbances, childhood-onset obesity, and peripheral adiposity (4).

Although this adverse phenotype, obese children and adolescents with PWS have a milder metabolic derangement compared to their BMI-matched controls (11, 45, 46). In particular, PWS children show lower insulin and C-peptide levels, lower insulin resistance, as well as higher adiponectin and HDL cholesterol levels (47). Our study confirmed these peculiarities: in patients with PWS, in fact, we found significantly lower fasting and post-OGTT insulin levels, C-peptide and HOMA-IR together with higher levels of adiponectin with respect to controls matched for BMI SDS, confirming better insulin sensitivity. In addition, HDL cholesterol levels in PWS patients appear to be slightly higher, also not significantly, than in controls.

Irisin, a myokine that induces the browning of WAT and regulates the transcription of thermogenic genes (16), has recently attracted attention for its potential role in obesity and metabolic syndrome *via* regulation of WAT accumulation and body weight control (48).

In children, the direction of the relationship between irisin levels and obesity is debated (25), with some authors reporting unaltered or even decreased irisin levels (28, 48), and others showing higher irisin levels in obese children as compared with healthy controls (28, 35, 49). Potential reasons for such discrepancy involve the role of peripubertal development on the interplay between fat mass, muscle, and fat/muscle mass ratio (25), as well as the potential for irisin to be secreted as a myokine in condition of healthy body weight (16, 50, 51) and as an adipokine in condition of fat accumulation (17, 20, 23).

In PWS, irisin has been primarily studied in adults and its levels have been reported to be lower than in BMI-matched obese controls, while being similar to those observed in normal weight subjects (20, 38, 39). In our population, we found that obese PWS children had lower irisin levels than controls with common obesity, a finding that parallels our previous results in PWS adults (20). Although our study confirmed significant differences in lean mass even in young PWS subjects as compared to their control counterpart, the lack of relationship between irisin and lean mass suggests the negligible role for the latter to explain differences in circulating irisin between groups. In a gender based analysis, irisin concentrations did not appear to differ between genders both in PWS and controls. While previous studies in children found higher irisin levels in girls, either lean or obese, than boys (22, 30), this difference has not been confirmed in subsequent studies in overweight or obese children (26, 35, 37). Analysis by the pubertal stage showed no difference in irisin levels between prepubertal and postpubertal children with PWS and in controls, which agrees with results from previous studies (28, 52). In particular, pediatric PWS with DEL15 have significantly reduced levels of irisin compared with controls with common obesity. Our results obtained in a young and homogenous populations, confirm previous finding by Faienza at al., who highlighted a potential role for PWS genotype, particularly DEL15 genotype, in decreasing irisin levels significantly in comparison to controls (40). Also, it is to be noted that a previous study showed that different genetic subtypes in PWS had also different endocrine characteristics, for instance growth hormone secretion (15).

Noteworthy, irisin partakes in the regulation of glucose homeostasis and insulin sensitivity by promoting glucose uptake and glycogenolysis, and by reducing gluconeogenesis (18, 34). In children and adolescents, many but not all authors described positive associations between irisin and insulin or glucose levels, and insulin resistance (26,31, 35, 53). In PWS and non-PWS children, our results support evidence of a significant positive association between circulating irisin level and several measures of insulin metabolism, including levels of levels and post-OGTT insulin, fasting peptide-C, and HOMA-IR and expand to the pediatric age previous observations obtained in adults with PWS (20). Insulin OGGT_120_ was the only predictor of irisin levels in the groups as a whole, suggesting a tight and independent association between irisin and post-absorptive insulin secretion. Noticeably, this relationship remained significant even after adjustment for potential confounders such as age, gender, and BMI, confirming previous similar observations (54). Based on evidence that irisin acts as an insulin-sensitizing hormone, facilitates liver and muscle glucose metabolism, and promotes β-cell survival (55), it is possible that irisin reflects a compensatory response to insulin resistance and initial deterioration of glucose tolerance. As such, divergences in irisin between PWS and non-PWS subjects reflect differences in insulin sensitivity, as confirmed by divergences in levels of adiponectin, an adipokine known for its insulin-sensitizing effects (10). Further, Reinher and coworkers reported higher irisin level in obese children with impaired glucose tolerance compared to obese children with normal glucose tolerance and normal-weight children (31). This implies that the lower irisin levels seen in PWS reflects their healthier glucose homeostasis as compared to obese controls, while the association documented between irisin and post-OGTT insulin levels could reflect its association with hyperinsulineamia and insulin resistance. Alternatively, Sesti et al. hypothesized that a decrease in insulin clearance could be the mechanism underlying higher irisin levels in individuals with increasing post-OGTT insulin levels (54), in the attempt to reduce β-cell stress due to increasing insulin resistance (53). Whether these mechanisms are responsible for current observations in children with and without PWS warrant further investigation. Together, we hypothesize that irisin is an active participant in the regulation of insulin resistance rather than an innocent by-stander. It is conceivable that irisin secretion increases in obesity to maximize energy usage and glucose homeostasis, so as to achieve metabolic balance and compensate for the underlying irisin resistance (18, 56).

Some limitations should be acknowledged in our study. First, it was based on cross-sectional analysis and so it is not possible to provide information on changes in irisin levels possibly linked to modifications of clinical parameters such as weight and metabolic balance, therefore, no conclusion regarding cause–effect relationships can be made. The HOMA model is only an assessment of insulin resistance. Clamp studies are actually the “gold standard” for analyzing insulin resistance. However, validation studies demonstrated a good correlation between HOMA and clamp techniques in young participants (57).

Notwithstanding these limitations, this study enrolled a consistent sample of PWS children that were extensively characterized in terms of anthropometric, clinical and metabolic profiling. Also, irisin assessment was carried out with what is currently considered as the best available irisin immunoassay (18).

Together, our findings suggest that circulating irisin levels are lower in PWS compared to BMI-matched children possibly due to differences in body composition and insulin resistance. The potential influence of a genetic component associated with PWS cannot be, however, entirely excluded and needs further assessment.

## Data Availability Statement

The raw data supporting the conclusions of this article will be made available by the authors, without undue reservation.

## Ethics Statement

The studies involving human participants were reviewed and approved by Ethical Research Committees of the Bambino Gesù Children’s Hospital. Written informed consent to participate in this study was provided by the participants’ legal guardian/next of kin.

## Author Contributions

Conceptualization, SM and PM; methodology, SM, RV, AlC, and SB; formal analysis, SM and PM; investigation, SM and DF; resources, DF, AnC, and MS; data curation, DF, SB, AlC; writing—original draft preparation, SM; writing—review and editing, DF, CM, MS, GG, GA, AnC, and PM; supervision, PM and MS. All authors have read and agreed to the published version of the manuscript.

## Funding

Research funded by the Italian Ministry of Health.

## Conflict of Interest

The authors declare that the research was conducted in the absence of any commercial or financial relationships that could be construed as a potential conflict of interest.

## Publisher’s Note

All claims expressed in this article are solely those of the authors and do not necessarily represent those of their affiliated organizations, or those of the publisher, the editors and the reviewers. Any product that may be evaluated in this article, or claim that may be made by its manufacturer, is not guaranteed or endorsed by the publisher.
